# Caution: Chemical Instability of Natural Biomolecules During Routine Analysis

**DOI:** 10.3390/molecules25143292

**Published:** 2020-07-20

**Authors:** Anaïs Pannequin, Erik Laurini, Laurent Giordano, Alain Muselli, Sabrina Pricl, Aura Tintaru

**Affiliations:** 1 UMR CNRS 6134 SPE, Laboratoire Chimie des Produits Naturels (CPN), Campus Grimaldi, Université de Corse, BP 52, 20250 Corte, France; pannequin_a@univ-corse.fr (A.P.); muselli_a@univ-corse.fr (A.M.); 2 Molecular Biology and Nanotechnology Laboratory (MolBNL@UniTS), DEA, University of Trieste, 34127 Trieste, Italy; erik.laurini@dia.units.it (E.L.); sabrina.pricl@dia.units.it (S.P.); 3 Aix Marseille Univ, CNRS, Ecole Centrale de Marseille, Institut des Sciences Moléculaires de Marseille UMR7313, 13397 Marseille, France; laurent.giordano@centrale-marseille.fr; 4 Department of General Biophysics, Faculty of Biology and Environmental Protection, University of Lodz, 90-236 Lodz, Poland; 5 Aix Marseille Univ, CNRS, Institut de Chimie Radicalaire UMR7273, 13397 Marseille, France

**Keywords:** active molecules, bryophytes, tamariscol, conocephalenol, artifacts, NMR, computational chemistry

## Abstract

Natural products (NPs) constitute a significant source of active biomolecules widely used in medicine, pharmacology and cosmetics. However, NPs structural characterization has the drawback of their chemical instability during the extraction steps and their likely transformation during the analytical protocol. In particular, tamariscol and conocephalenol are two compounds largely used in the cosmetic industry for their odorant properties. Thus, in the present study, we focused on the evolution of these two metabolites (extracted from *Frullania tamarisci* and *Conocephalum conicum,* respectively), as followed by NMR. Interestingly, we found that, once dissolved in deuterated chloroform, these two tertiary alcohols are both subjected to transformation processes, leading to degradation compounds with altered structures. Accordingly, these detected degradation compounds have been fully characterized by NMR and the experimental findings were supported by computational chemistry data.

## 1. Introduction

Nowadays, natural products (NPs) represent an important source of new active compounds largely used in biological, aromatherapy, and cosmetic applications. One of the main problems encountered during their manipulation and study is linked to their chemical instability; this has a significant impact both on their initial detection/identification and on the accuracy of their analytical characterization. Many natural biomolecules and metabolites are highly reactive compounds; consequently, they can potentially lead to artifacts’ formation during their isolation, purification and characterization [1]. In his review [1], Hanson reported a list of the main mechanisms often resulting in artifacts, which include dehydration [2], rearrangement [3] and oxidation reactions [4]. In addition, once in contact with organic solvents, NPs may be subjected to chemical transformations which can alter their odorous, appearance, and even biological properties [5,6]. Most of these processes are related to thermal factors; therefore, the composition of essential oils obtained via supercritical carbon oxide extraction is often reported to be different from those obtained using the traditional method of steam distillation [7,8]. Hanson [1] also summarizes the formation of artefacts due to the solvent used during compound isolation. As an example, in the case of *Scutelaria discolor*, the used of EtOH will lead to the formation of an acetal [9]. Analogous artefact formation could be observed in the study reported by Wang et al. [10]

Although the formation of artifacts during the isolation of natural metabolites could be a cause of error in NP studies, it can nevertheless provide useful chemical information once the unexpected transformation has been recognized. For example, some degradation products can exhibit pharmacological effects, as demonstrated for curcumin by-products, which exhibited promising activity against important pathologies such as Alzheimer’s disease and cancer [11,12]. Therefore, artifacts formation remains an important issue and their identification/characterization constitute a major milestone in the chemistry of NPs. Nevertheless, literature data are not very informative on the eventual degradation effects appearing during NPs analysis involving different experimental methodologies. In particular, nuclear magnetic resonance (NMR)—a technique indispensable to any molecular characterization—has the great disadvantage of long acquisition times compared to other spectroscopic techniques (e.g., infrared (IR) or UV-visible spectroscopy, or mass spectrometry (MS). Such extended contact times between solvent and compounds might indeed induce structural alterations in the molecules under investigation.

**Figure 1:**
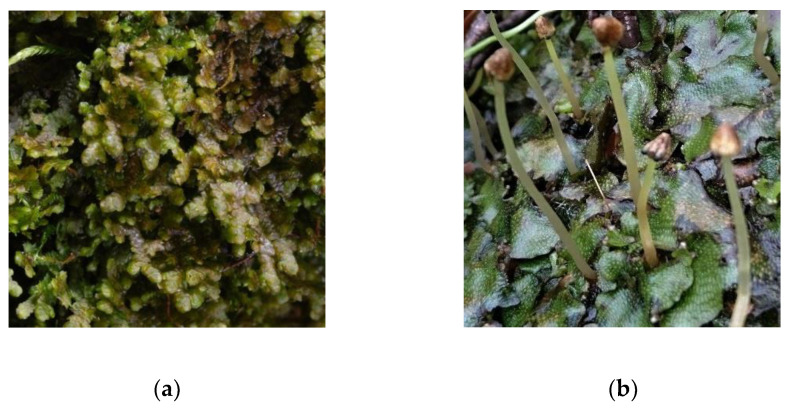
(**a**) Leave of *Frullania tamarisci* and (**b**) thalli of *Conocephalum conicum*.

Under this perspective, in the current work, we considered the evolution of tamariscol and conocephalenol, two sesquiterpene alcohols and main components of the liverworts *Frullania tamarisci* [13] and *Conocephalum conicum* [14], respectively (Figure 1), during their NMR characterization.

**Scheme 1:**
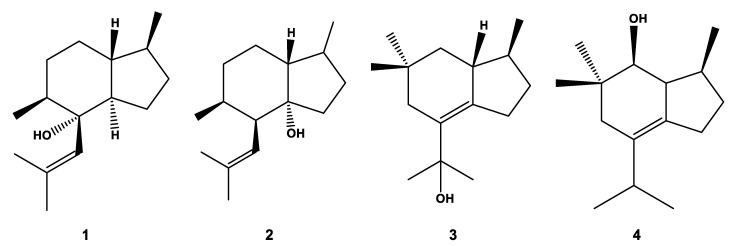
Structures of tamariscol (**1**), pacifigorgiol (**2**), conocephalenol (**3**) and brasilenol (**4**).

Tamariscol (**1**, Scheme 1) is mainly mentioned as a constituent of European [13] and Japanese [15] leafy *Frullania tamarisci* liverwort. Since its first structural description in 1984 [13], this sesquiterpene alcohol and its corresponding epoxide are largely used in cosmetics due to their strong mossy odor, priceless for the perfume industry. Different synthesis methods have been proposed and allowed for the determination of the tamariscol stereochemistry [16,17,18]. Tamariscol exhibits a rare pacifigorgiane carbon skeleton as pacifigorgiol (**2**, Scheme 1), another sesquiterpene alcohol isolated from corals [19]. Interestingly, both **1** and **2** may undergo dehydration in the presence of pyridine and SOCl_2_ leading to different pacifigorgiane derivatives [20]; however, no clear and complete analytical description has been reported in the current literature accounting for a solvent-induced transformation of these two natural compounds.

*Conocephalum conicum* is a thalloid liverwort worldwide spread which thalli emanate a turpentine odor when squeezed. Conocephalenol (**3**, Scheme 1) is also a natural sesquiterpene tertiary alcohol and represents one of the main components of *C. conicum* extracts [14]. It possesses an unusual molecular skeleton analogous to the one characterizing brasilenol (**4**, Scheme 1), another irregular sesquiterpene alcohol extracted from red algae (*Aplysia brasiliana*) and marine mollusks (*Laurencia obtusa*) [21,22]. In analogy with **1** and **2**, three brasiladiene derivatives were reported to be formed upon conocephalenol dehydration when treated with POCl_3_. However, the reported studies assess the presence of those products in the natural mixture independently of the preparation method, excluding their formation during the preparation of the extracts [23].

In order to gain original data and a deeper understanding of the possible solvent-induced structural modifications of NPs, we decided to monitor tamariscol and conocephalenol chemical stability in the presence of deuterated chloroform during their NMR analytical step as a proof-of-concept. On the one hand, both **1** and **3** feature a bicyclo[4.3.0]nonane skeleton, but their particularity consists of a tertiary alcohol moiety suitable to undergo dehydration and generate artifact formation. On the other hand, commercial CDCl_3_, which constitutes the widest used organic solvent for NMR studies of NPs, contains a small amount of CHCl_3_, often known as the residual [24]. This, in turn, could be subjected to degradation effects and formation of low quantities of HCl [5], and the so-induced acidity could ultimately generate artifact compounds within the natural mixture [25]. Since the good knowledge of the chemical stability of NPs represents an important milestone in the field of phytochemistry, in the remainder of this paper we report and discuss the results of a combined experimental and theoretical study focused on the characterization of the degradation compounds observed when natural samples mainly containing either tamariscol or conocephalenol were submitted to routine NMR analysis in presence of CDCl_3_. The analyzed samples were obtained upon the fractionation of essential oils from *F. tamarisci* and *C. conicum*, exclusively prepared from plants harvested on Corsica Island.

## 2. Results and Discussion

### 2.1. Tamariscol Investigation

**Scheme 2:**
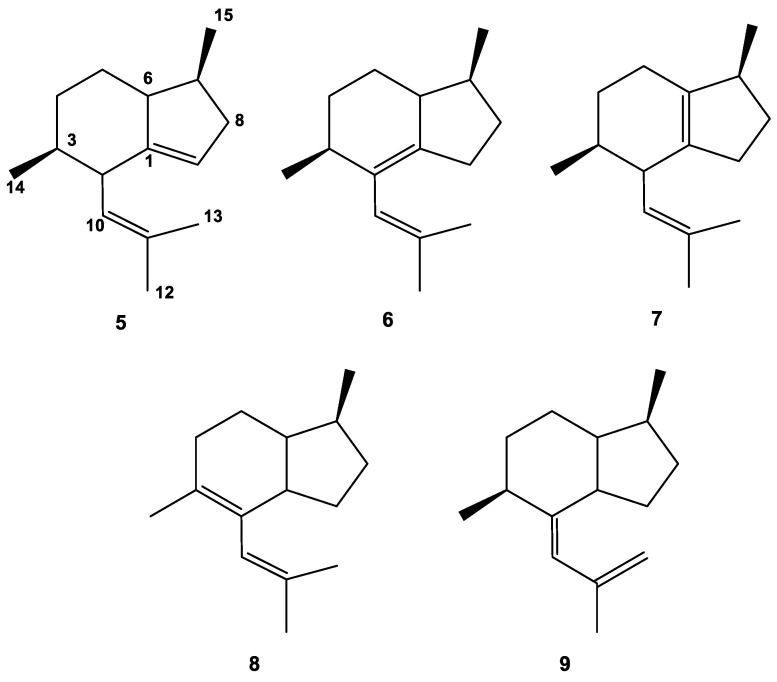
Structures of pacifigorgia-1(9),10-diene (**5**), pacifigorgia-1,10-diene (**6**), pacifigorgia-1(6),10-diene (**7**), pacifigorgia-2,10-diene (**8**) and pacifigorgia-2(10),11-diene (**9**).

Before reporting the results obtained while studying tamariscol (**1**), it is important to note that, although mentioned as the main constituent of many *Frullania* liverworts, Paul et al. reported the absence of **1** in *F. fragilifolia* essential oils [20] which, in contrast, contained five different pacifigorgianes: pacifigorgia-1(9),10-diene (**5**), pacifigorgia-1,10-diene (**6**), pacifigorgia-1(6),10-diene (**7**), pacifigorgia-2,10-diene (**8**) and pacifigorgia-2(10),11-diene (**9**) (Scheme 2). Contextually, the same authors reported the presence of tamariscol, as well as of **5**, **7**, **8**, and **9** in *F. tamarisci* essential oils, with the remarkable absence of pacifigorgia-1,10-diene (**6**).

**Figure 2:**
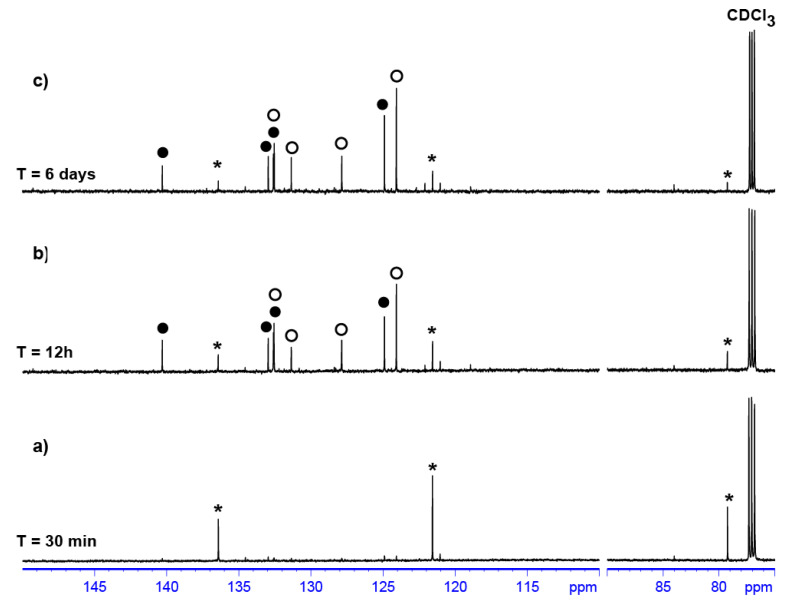
Partial ^13^C-NMR spectra (region between 150 and 75 ppm) of the *F. tamarisci* essential oil fraction enriched in tamariscol (>95%), acquired in CDCl_3_ (150.90 MHz, 300K) at (**a**) 30 min, (**b**) 12 h and (**c**) 6 days respectively, after sample preparation (signals annotation refers to compounds **1** (*), **6** (●) and **8** (○), respectively). For the sake of clarity, only the interval of chemical shifts between 150 and 75 ppm, characteristic for the ethylene resonances and C-O bonds, respectively, are shown. The whole ^13^C NMR spectra are reported in Figure S1.

In our previous work, upon analysis of 12 essential oils prepared from Corsican *F. tamarisci*, tamariscol was reported as the main component, and it was detected with concentration varying between 26.3 to 41.5% [26]. Additionally, ^13^C-NMR spectra recorded on oil-fractions of *F. tamarisci* obtained by column chromatography revealed the co-elution of tamariscol with pacifigorgiol (**2**) in GC-FID and GC-MS, both on apolar and polar columns [26]. To date, **2** was found only in a Pacific coral, *Pacifigorgia adamsii* [19] and a vascular plant, *Valeriana officinalis* [20]. Moreover, upon GC-FID and GC-MS analysis of our Corsican samples, the presence of some pacifigorgianes was also indubitably assessed (Table S2), while compounds **5**, **7** and **9** were straightforwardly identified by NMR based on literature data [20]. Nevertheless, we tentatively monitored by ^13^C-NMR the evolution of an oil-fraction containing 95% tamariscol upon dissolution in CDCl_3_. The recorded chemical shift values—in full agreement with literature reports (Figure 2a)—were assigned to the targeted sesquiterpene alcohol **1 [13]**. Quite remarkably, no traces of pacifigorgiol (**2**) or of other pacifigorgianes (Scheme 2) were detected in our analyzed sample.

Several hours later, however, a newly acquired ^13^C-spectrum showed the decrease of NMR signals corresponding to **1** and the concomitant appearance of supplementary resonances, which could not be unequivocally assigned. However, the values of these further chemical shifts suggested the presence of new pacifigorgiane skeletons, likely attributable to compounds originating from the dehydration of **1** (Figure 2b). The subsequent structure assignment performed on the bases of the corresponding 1D- and 2D-NMR data (see Experimental section for the complete data set) led to the conclusions that both newly formed molecules represent two isomeric forms issued from the dehydration reaction of **1**, i.e., pacifigorgia-1, 10-diene (**6**) and pacifigorgia-2, 10-diene (**8**) (Scheme 2). In support of this, both these two pacifigorgianes have already been reported as degradation products of tamariscol formed in the presence of pyridine and SOCl_2_, although their structures were characterized solely on the basis of ^1^H-NMR data [20]. In addition, a third, new ^13^C-NMR spectrum acquired after few days revealed the further increase of these pacifigorgiane resonances in parallel with the drastic decrease of tamariscol signal intensities (Figure 2c), leading us to conclude that the dehydration reaction of the tamariscol indeed takes place in CDCl_3_. Moreover, the so-formed compounds **6** and **8** are found to be very stable, and their detection is still feasible also after a few weeks’ storage at the room temperature (data not shown). Accordingly, these results therefore represent a good example where a natural compound could evolve during storage and produce molecular entities, which are also reported as metabolites of the crude vegetal material.

**Scheme 3:**
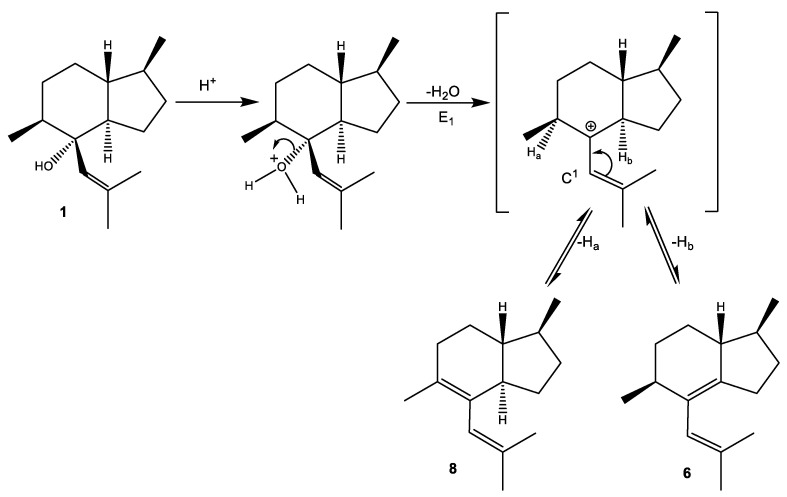
Proposed dehydration and rearrangement mechanisms of tamariscol (**1**) in presence of CDCl_3_ at room temperature, leading to the formation of the dienic derivatives **6** and **8** formation.

At this point, it is important to mention that the molecular configuration of **1** in the natural product, as experimentally determined by NMR in chloroform solution [13,17,18], presents the OH substituent of the cyclohexane moiety in the equatorial position. Such configuration prevents water elimination following E2 mechanism, because the atoms are not in the antiperiplanar configuration as required [27], while a dehydration reaction can take place when the same OH group occupies an axial position. This assumption oriented us toward an E1 elimination, which would imply the formation of an intermediate carbocation. To investigate this aspect in more detail, we resorted to computational chemistry techniques. According to our quantum chemistry-based calculations performed on tamariscol using CDCl_3_ as a solvent, the most stable conformer of **1** in a CDCl_3_ solution features indeed the -OH group in the equatorial position with the hydrogen atom of this unit oriented in a *trans* configuration with respect to the axial alkene chain. Therefore, we assessed the dehydration reaction mechanism as proposed in Scheme 3, starting from tamariscol in this conformation (Table 1), following a classical E1 pathway. The relevant results are reported in Table 1, together with the corresponding optimized structures of **1**, **6**, **8** and **C^1^**.

This Table shows that the generation of the carbocation **C^1^** from **1** (Scheme 3) requires the crossing of the energy barrier ΔG**_C_^1^_−1_** = +1.775 kcal/mol. Then, the subsequent formation of both compounds **6** and **8** is thermodynamically favored, with a negative variation of the corresponding free energy values of ΔG**_6–C_^1^** = −1.971 kcal/mol and ΔG**_8–C_^1^** = −2.264 kcal/mol, respectively. Accordingly, the total free energy changes involved in the dehydration reaction of tamariscol leading to pacifigorgia-1, 10-diene (**6**) and pacifigorgia-2, 10-diene (**8**) are also quite thermodynamically favored, i.e., ΔG**_6–1_** = −0.196 kcal/mol, and ΔG**_8–1_** = −0.489.

Overall, these computational results suggest that the relatively high energetic barrier ΔG**_C_^1^_-1_** is one of the main factors contributing to the slow kinetics of tamariscol dehydration reaction observed in CDCl_3_. From the thermodynamic standpoint, however, the reaction pathway leading from **1** to **6** and **8** via the carbocation **C^1^**—as proposed in Scheme 3—is a spontaneous process in CDCl_3_ at room temperature, in agreement with the corresponding experimental evidence (Figure 2).

### 2.2. Conocephalenol Investigation

**Figure 3:**
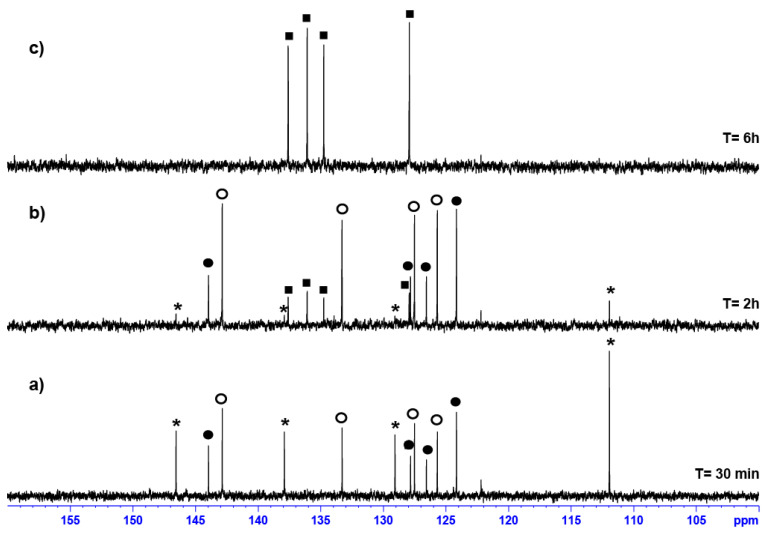
Partial ^13^C-NMR spectra (region between 160 and 100 ppm) of conocephalenol rich fraction (80%) acquired in CDCl_3_ (150.90 MHz, 300K) at (**a**) 30 min, (**b**) 2h and (**c**) 6h respectively, after sample preparation (N.B. signal annotation refers to compounds **10** (*), **11** (●), **12**(○) and **13** (■), respectively). For the sake of clarity, only the interval of chemical shifts between 160 and 100 ppm, characteristic for the ethylene resonances, are shown. The whole ^13^C NMR spectra are reported in Figure S2).

Conocephalenol (**3**, Scheme 1) presents some structural similarity with **1** because of its bicyclo[4.3.0]nonane skeleton and the tertiary alcohol function that can also be subjected to dehydration reaction, leading to diene formation. Therefore, **3** constitutes another good candidate to be investigated by NMR upon dissolution in CDCl_3_. In this context, we have analyzed samples issued from the fractionation of two essential oils and two diethyl ether extracts prepared from *C. conicum*, in which **3** represents up to 19% of the chemical composition (Table S3). So far, the available literature data only report NMR analysis of conocephalenol in deuterated benzene [28], the reason for this likely residing in the compound’s molecular fragility in CDCl_3_. As mentioned in the introduction, and as discussed above for tamariscol, the small amount of HCl originating from the presence of CHCl_3_ in commercial CDCl_3_ NMR solvent may induce artifacts formation within the conocephalenol-based NP mixture. In order to investigate the degradation mechanism of **3**, we again employed a solvent-extract fraction enriched in the NP under investigation (80%). A part of this sample has been firstly dissolved in C_6_H_6_ and we acquired the relevant ^13^C-NMR spectrum in order to confirm the presence of the desired structure (data not shown). The remaining fraction was next dissolved in CDCl_3_ and the corresponding ^1^H- and ^13^C-NMR spectra were acquired immediately after sample preparation. As seen in Figure 3a, the ^13^C-NMR spectrum showed peaks related to the presence of brasila-5,10-diene (**10**) along with small amounts of brasila-1(6),5(10)-diene (**11**) and brasila-5(10),6-diene (**12**) (Scheme 2) in almost equivalent ratios. Quite remarkably, the spectrum did not exhibit any resonance attributable to conocephalenol.

Two hours later, a new ^13^C-NMR spectrum recorded from the same sample (Figure 3b) revealed that the resonances attributable to compound **10** completely disappeared, while the remaining signals reflect the presence in solution of a mixture of **11** and **12** in an approximate ratio of 1:2. Most surprisingly, however, some new peaks could also be located between 150 and 100 ppm, supporting the appearance of new species containing sp^2^ carbons. Experiments were therefore repeated 6 h later (again on the same sample at room temperature) and the relevant ^13^C- spectrum (Figure 3c) disclosed the presence of signals attributable to a completely new compound - brasila-1(9),5-diene (**13**) - which, to the best of our knowledge, has never been reported to date, either as natural or artifact product. We then confirmed the final structure of **13** by 2D NMR data, and this is shown, together with its full ^1^H- and ^13^C-NMR peak assignment, in Table 2.

**Scheme 4:**
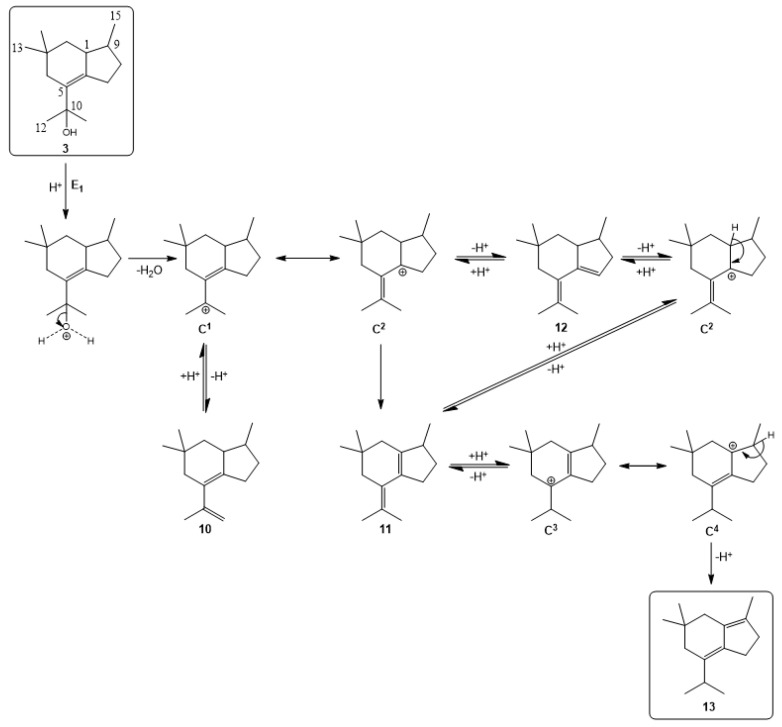
Proposed dehydration and rearrangement mechanisms of conocephalenol (**3**) in presence of CDCl_3_ at room temperature, leading to the formation of the dienic derivatives **10**, **11**, **12**, and **13**. Note that carbocations **C^1^**–**C^2^** and **C^3^**–**C^4^** represent mesomeric forms, respectively.

We reasoned that, within the entire series of diene derivatives which could be formed upon the dehydration of the sesquiterpene alcohol **3**, **13** would be the most stable, due to hyperconjugation effects. This assumption would therefore support the evolution of **10**, **11** and **12** toward **13**. Moreover, the presence of an exocyclic double bond should endow **10** with the lowest stability, again supporting its rapid transformation into the other brasiladiene derivatives. In line with this, Scheme 4 thus illustrates the sequence of phenomena observed during the analysis of conocephalenol sample prepared in deuterated chloroform.

In order to verify the validity of our assumptions and the feasibility of the reaction mechanisms as laid out in Scheme 4, we resorted again to computational chemistry. Accordingly, Table 3 shows the optimized structures of **3**, **10**, **11**, **12**, **13**, **C^1^**, **C^2^**, **C^3^** and **C^4^** (Scheme 4), along with the corresponding calculated energy data.

As seen from this Table, the calculated energy of conocephalenol in CDCl_3_ at room temperature (E_tot_ = –585.52766 Ha, G = –26.091 kcal/mol) is not very different from that of **10** (E_tot_ = –585.52770 Ha, G = –26.113 kcal/mol), i.e., the first compound resulting from the dehydration reaction of **3** according to the proposed reaction pathways (Scheme 4). Using the values in Table 3, the energy change involved in each reaction step leading from **3** to **10** via the formation of the intermediate carbocation **C^1^** can be easily calculated as follows. First, to form **C^1^** from **3**, the system must overcome a very small energetic barrier ΔG**_C_^1^_–3_** equal to +0.233 kcal/mol. Then, the generation of **10** from **C^1^** is a thermodynamically spontaneous process, as the corresponding ΔG**_10–C_^1^** value amounts to –0.255 kcal/mol. Accordingly, the overall change in free energy for the conversion of **3** to **10** (ΔG**_10–3_**) is negative (i.e., favorable) and equal to –0.022 kcal/mol. As soon as **10** rapidly forms from **3** in the solution, Scheme 4 and the values in Table 3 can further explain the remaining cascade of reactions, ultimately resulting in the formation of the new compound **13**. Thus, according to Scheme 4, **12** can be formed from **10** via the two carbocations **C^1^** and **C^2^**. Using the relevant energy values in Table 3, we can estimate that i) **10** can quickly generate the intermediate carbocation **C^1^** by easily crossing the relevant low energy barrier at room temperature (ΔG**_10–C_^1^** = +0.255 kcal/mol), ii) **C^1^** can interconvert into its mesomeric form at no energy expenses (ΔG**_C_^1^_–C_^2^** = 0 kcal/mol), and iii) **12** can finally form from **C^2^** with a ΔG**_12–C_^2^** of -0.508 kcal/mol. Therefore, the formation of **12** from **10** is thermodynamically favored, with a negative variation of the corresponding free energy of ΔG**_12–10_** = -0.253 kcal/mol. Following the proposed reaction scheme further, compound **11** can be originated either from **10**—via a pathway utterly similar to that leading to **12**—or from **12** (once it has been generated from **10**) via the indicated rearrangement of the **C^2^** cation (Scheme 4). Concerning the first mechanism, the relevant energy values listed in Table 3 indicate that, while the first two steps coincide with those described above for the reaction leading to **10** from **12** (i.e., ΔG**_10–C_^1^** = +0.255 kcal/mol and ΔG**_C_^1^_–C_^2^** = 0 kcal/mol), the free energy difference for the conversion of **C^2^** to **11** is ΔG**_11–C_^2^** = –0.734 kcal/mol. Therefore, the formation of **11** from **10** is also thermodynamically favored, with a negative total free energy variation (ΔG**_11–10_**) of –0.479 kcal/mol. Contextually, to obtain **11** from **12** via the second mechanism (Scheme 4), first the small energy barrier ΔG**_C_^2^_–12_** = -ΔG**_12–C_^2^** = +0.508 kcal/mol) must be overcome to generate the carbocation **C^2^**, followed by the thermodynamically favorable conversion of **C^2^** into **11** (ΔG**_C_^2^_–11_** = −0.734 kcal). Overall, this pathway is also favored with a total ΔG**_11–12_** of –0.226 kcal/mol. Finally, the new compound **13** can be obtained from **11**, first via i) the generation of the carbocation **C^3^** (ΔG**_C_^3^_–11_** = +0.838 kcal/mol), ii) the isoenergetic formation of the mesomeric cation C^4^ (Δ**G_C_^4^_–C_^3^** = 0 kcal/mol), and iii) the final generation of **13** from **C^4^** (ΔG**_13–C_^4^** = −2.017 kcal/mol). Overall, the formation of **13** from **11** is quite thermodynamically favored, as the corresponding variation in free energy ΔG**_13-11_** is equal to −1.179 kcal/mol. In summary, the computational results discussed above support the experimental evidence of a series of possible spontaneous transformations—in CDCl_3_ and at room temperature—leading from **3** to the newly reported compound **13**, along the reaction pathways laid out in Scheme 4.

In parallel, we scrutinized additional samples of conocephalenol fractions prepared from natural extracts, as obtained by extraction in di-ethyl ether, hydrodistillation and solid phase microextraction (SPME). Some brasiladienes could also be detected after GC-MS and GC-FID analysis of the natural mixtures (Table S3). These results assess the presence of **10**, **11** and **12** also as constituents of the natural mixture; in the same way, our study underlines their formation upon dehydration of the conocephalenol. In this context, we might assume that the presence of the brasiladienes in the natural mixture could be a result of the enzymatic dehydration of the conocephalenol into the vegetal cells; this phenomenon would explain their detection in all the analyzed samples, independently of the technics used for its preparation. However, independently of the extraction method, compound **3** has never been detected after the dissolution of the natural mixture in normal CDCl_3_. In order to prove the degradation effect due of the acidity of the solvent, a fraction containing conocephalenol has been dissolved in CDCl_3_ previously filtered on the basic alumina powder, which should neutralize all the acidic traces. Pleasingly, the new ^13^C spectrum recorded on this last sample exhibited all expected conocephalenol resonances, in agreement with those obtained in C_6_H_6_ (Figure S3), and as further confirmed by 2D-NMR correlation data.

Therefore, we could conclude that, as in the case of tamariscol, the dehydration process of conocephalenol is also related to small quantities of acidic species present in the NMR solvent; in contrast, however, the kinetics of water loss reaction is much faster in the case of **3** as compared to **1**. This can be related to the fact that the OH group of conocephalenol is free from steric hindrance, being situated at the extremity of the isopropyl substituent, and to the relatively low energy difference between **3** and its first degradation product **10**, as verified by quantum chemistry calculation. Accordingly, the corresponding dehydration reaction can take place along a very smooth energetic pathway, involving simple and low-energy molecular rearrangements.

## 3. Materials and Methods

### 3.1. Chemicals

Deuterated chloroform (99.98% D) and benzene -D6 (99.90% D) were purchased from Sigma Aldrich (St. Louis, MO, USA). All chemicals were used as received without further purification.

### 3.2. Nuclear Magnetic Resonance

NMR spectra were recorded on different essential oil fractions dissolved in CDCl_3_ or C_6_H_6_ (EuroIsotop. Saint Aubin, France) at 300K, using a Bruker Avance III NMR spectrometer (Bruker, Karlsruhe, Germany), operating at 600.13 MHz for ^1^H and 150.90 MHz for ^13^C Larmor frequency, with double resonance broadband fluorine observation (BBFO) 5 mm probe head. ^13^C-NMR experiments were recorded using one-pulse excitation pulse sequence (90° excitation pulse) with ^1^H decoupling during signal acquisition (performed with WALTZ-16); the relaxation delay has been set to 2s. For each sample analyzed, depending on the compound concentration, 1k scans (50 min acquisition time) up to 3k scans (approx. 2.5 h) and 64k complex data points were collected using a spectral width of 30,000 Hz (240 ppm). Chemical shifts (δ in ppm) were reported relative to the residual signal of CDCl_3_ (δ_C_ 77.04 ppm). Complete ^1^H and ^13^C assignments of the new degradation compounds observed for the conocephalenol sample (**6** and **8**, see below) were obtained using 2D gradient-selected NMR experiments. ^1^H-^1^H COSY (COrrelation SpectroscopY), ^1^H-^13^C HSQC (heteronuclear single quantum correlation), ^1^H-^13^C HMBC (heteronuclear multiple bond coherence) and ^1^H-^1^H NOESY (nuclear overhauser effect spectroscopy), for which conventional acquisition parameters were used as described in the literature [29]. Full NMR assignment of pacifigorgia-1,10-diene (**6**), ^13^C (ppm) at 150.90 MHz, 300 K in CDCl_3_ (77.04 ppm): 140.32 (C-2), 132.97 (C-11), 132.56 (C-1), 124.90 (C-10), 49.10 (C-6), 41.84 (C-7), 34.21 (C-3), 33.00 (C-4), 32.99 (C-8), 27.75 (C-9), 27.72 (C-5), 25.31 (C-12), 21.26 (C-14), 19.80 (C-13), 18.26 (C-15); ^1^H (ppm) at 600.13 MHz, 300 K in CDCl3 (7.26 ppm): 2.15 (H-3), 1.97/1.18 (H-4), 1.74/1.22 (H-5), 1.72 (H-6), 1.32 (H-7), 1.82/0.99 (H-8), 1.98/0.99 (H-9), 1.78 (H-12), 1.58 (H-13), 0.94 (H-14), 1.05 (H-15). Full NMR assignment of pacifigorgia-2,10-diene (**8**), ^13^C (ppm) at 150.90 MHz, 300 K in CDCl_3_ (77.04 ppm): 132.60 (C-11), 131.36 (C-3), 127.88 (C-2), 124.07 (C-10), 51.61 (C-6), 48.40 (C-1), 37.97 (C-7), 33.39 (C-8), 32.22 (C-5), 28.90 (C-4), 26.42 (C-9), 25.18 (C-12), 19.91 (C-14), 19.62 (C-13), 18.85 (C-15); ^1^H (ppm) at 600.13 MHz, 300 K in CDCl3 (7.26 ppm): 1.92 (H-1), 2.31/2.03 (H-4), 1.94/1.23 (H-5), 0.98 (H-6), 1.53 (H-7), 2.20 (H-8), 1.93/1.27 (H-9), 5.6 (H-10), 1.78 (H-12), 1.55 (H-13), 1.55 (H-14), 1.02 (H-15).

### 3.3. Plant Material, Essential Oil Isolation and Fractionation

Plant material: fresh F. tamarisci and *C. conicum* plants were harvested from different localities of Corsica (France), and voucher specimens were deposited in the herbarium of University of Corsica, Corte, France. Fresh F. tamarisci and *C. conicum* were harvested in three and five locations of Corsica (France), respectively. Botanical determination was performed according to the botanical determination keys summarized in Bryophyte Flora [30], and voucher specimens were deposited in the herbarium of University of Corsica (Corte, France). The sample numbers, the geographical origin of the different samples and the voucher codes for each analyzed specimen are listed in Table S1.

Essential oil isolation: the fresh plant material (300 g) was subjected to hydrodistillation (5 h) using a Clevenger-type apparatus, according to the method recommended in the European pharmacopoeia [31]. The essential oil yields (*F. tamarisci* 0.13–0.42% and *C. conicum* 0.14–0.24%) were expressed in % (*w*/*w*, based on the weight of the dried plant material).

Essential oil fractionations: S1 (June-15) and S3 sample oils (500 mg) of *F. tamarisci* and S2 (June-15) of *C. conicum* were submitted to column chromatography on a silica-gel column (200−500µm, 12 g, Clarisep^®^ Bonna Agela Technologies, Willington, DE, USA) with Combi Flash apparatus (Teledyne ISCO, Lincoln, NE, USA), equipped with a fraction collector monitored by an UV detector. Using gradients of (*v*/*v*) hexane/diisopropyl ether (HEX/DIPE), ten fractions (2 with hydrocarbons and 8 with the oxygenated compounds) were eluted from S1 and twenty-two (1 with hydrocarbons and 21 with the oxygenated compounds) were eluted from S3 of *F. tamarisci*, while eleven fractions (2 with hydrocarbons and 9 with oxygenated compounds) were eluted from S2 of *C. conicum*, respectively.

Sample storage: all samples were stored 8 °C prior to the analysis. Details of the essential oils preparation method are reported in our previous works [26,32].

### 3.4. Computational Chemistry

All calculations were carried out with Gaussian16 [33]. All molecular geometry optimization and the corresponding single point energy calculations were performed at the MP2/6-311++G(d,p) level in the presence of chloroform as a solvent (ε = 4.7113), using the polarizable continuum model (PCM) [34]. Molecular geometries corresponding to energy minima were identified by performing energy minimization with respect to all coordinates and without imposing any constraint. Vibrational frequency confirmed all analyzed structures were either stable energy minima (0 imaginary frequencies) or transition states (1 imaginary frequency) and yielded the corresponding zero-point vibrational energies (ZPVEs). The structures of the molecular transition-state geometries were located using the optimized geometries of the equilibrium molecular structures following the procedure of Dewar et al. [35].

## 4. Conclusions

The fragility of certain natural products, their intrinsic reactivity or their potential reaction with different solvent can promote the formation of molecular artifacts during, e.g., their analytical characterization. In this work we showed this phenomenon by considering two NP tertiary alcohols —tamariscol and conocephalenol—which undergo molecular degradation via a dehydration process catalyzed by HCl present in traces within the solvent used for their NMR analysis, namely CDCl_3_. It must be mentioned that some of the so-formed compounds are also detected as constituents of the natural mixture. Therefore, even if during the isolation, preparation and manipulation of natural extracts major attention is paid to the experimental conditions (temperature, pressure, etc.), the time required to acquire analytical data and/or short sample storage times can result in significant molecular transformation with consequently altered properties of the corresponding natural matrix. However, deuterated chloroform remains a good organic solvent, cheap and easy to use for NMR routine analysis. Its acidity is well known and proven; therefore, it can be avoided by systematically filtration on basic alumina prior to sample dissolution. As reported in the present study, the alteration of the natural sample can be observed at variable laps of time after sample preparation. Therefore, we warn the researchers on the fact that some compounds can easily evolve and potentially induce error in data interpretation. The structure alteration is likely to induce important changes on the physical properties of the compounds and drastically modify its biochemical virtues. Nevertheless, the biological properties of the formed artifacts should also be evaluated.

## Figures and Tables

**Table 1:** Optimized structures, energies, and thermodynamic functions (all corrected for ZPVE) for the optimized structures of **1**, **6**, **8**, and **C^1^** (Scheme 3) in solution (CDCl_3_) at room temperature. E_tot_ = total molecular energy (Ha, 1 Ha = 627.509 kcal/mol); H = H_298_ – H_0_ = enthalpy (kcal/mol), S = entropy (cal/mol K), G = G_298_ – G_0_ = Gibbs free energy (kcal/mol). In the molecular structures, C, O, and H atoms are shown as gray, red, and white spheres, respectively.

	E_tot_	H	S	G	
**1**	–662.12429	11.323	137.046	–29.517	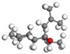
**6**	–662.12187	11.636	138.686	–29.713	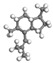
**8**	–662.12234	11.297	138.530	–30.006	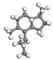
**C^1^**	–662.11873	12.701	135.647	–27.742	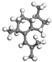

**Table 2:** 2D/3D structure and NMR data for brasila-1(9),5-diene **(13)** in CDCl_3_ (600.13 MHz, 300K). In the 3D structure, C and H atoms are shown as gray and white spheres, respectively.

	**Atom Number**	**δ(^13^C) ppm**	**Carbone Type ^a^**	**(^1^H) ppm**	**Multiplicity/** **J ^c^ (Hz)**	**HMBC ^b^**
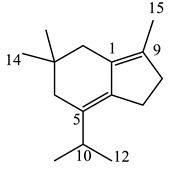	1	134.74	C=	-	-	H_2_, H_8_, H_13,14_, H_15_
	2	37.07	CH_2_	1.97	m	H_4_, H_13,14_
	3	30.23	C_q_	-	-	H_2_, H_4_, H_13,14_
	4	37.57	CH_2_	1.80	m	H_2_, H_10_, H_13,14_
	5	127.91	C=	-	-	H_4_, H_7_, H_10_, H_11,12_
	6	137.59	C=	-	-	H_2_, H_4_, H_7_, H_8_, H_10_
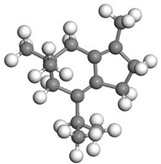	7	24.93	CH_2_	2.46	m	H_8_
	8	36.05	CH_2_	2.41	m	H_7_, H_15_
	9	136.07	C=	-	-	H_2_, H_7_, H_15_
	10	30.75	CH	2.62	hept, 6.88	H_4_, H_11,12_
	11, 12	20.13	CH_3_	0.98	d, 6.88	H_10_, H_11,12_
	13, 14	28.62	CH_3_	0.90	s	H_2_, H_4_, H_13,14_
	15	14.27	CH_3_	1.75	s	H_8_

^a^ Partial assignment established from Distortionless Enhancement by Polarization Transfer (DEPT) experiments (see and ^13^C-chemical shift tables. ^b^ Long-range correlations C→H are reported as extracted from HMBC data. ^c^ J = coupling constant.

**Table 3:** Optimized structures, energies and thermodynamic functions (all corrected for ZPVE) for the optimized structures of **3**, **10**, **11**, **12**, **13**, **C^2^**, **C^3^**, **C^4^** and **C^5^** (Scheme 3), in solution (CDCl_3_) at room temperature. Symbols and colors as in Table 2.

	E_tot_	H	S	G	
**3**	−585.52766	10.547	122.947	−26.091	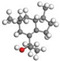
**10**	−585.52770	10.726	123.621	−26.113	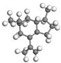
**11**	−585.52846	10.698	125.134	−26.592	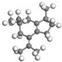
**12**	−585.52810	10.641	124.185	−26.366	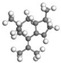
**13**	−585.53034	11.232	130.883	−27.771	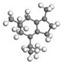
**C^1^**	−585.52729	10.510	122.042	−25.858	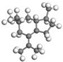
**C^2^**	–585.52731	10.513	122.050	–25.858	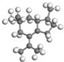
**C^3^**	–585.52713	10.495	121.642	–25.754	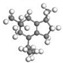
C^4^	–585.52711	10.491	121.627	–25.754	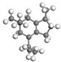

## Supplementary Materials

The following are available online at https://www.mdpi.com/1420-3049/25/14/3292/s1: ^1^H and ^13^C NMR data of analyzed compounds (Figures S1, S2 and S3), Chemical composition of natural extracts (Tables S1, S2 and S3).

## References

[1] Hanson J.R. (2017). Pseudo-Natural Products, Some Artefacts Formed during the Isolation of Terpenoids. J. Chem. Res..

[2] Weston R.J. (1984). Composition of essential oil from leaves of Eucalyptus delegatensis. Phytochemistry.

[3] De Kraker J.-W., Franssen M.C.R., De Groot A., Shibata T., Bouwmeester H.J. (2001). Germacrenes from fresh costus roots. Phytochemistry.

[4] Toyota M., Koyama H., Mizutani M., Asakawa Y. (1996). (−)-ent-spathulenol isolated from liverworts is an artefact. Phytochemistry.

[5] Maltese F., Van Der Kooy F., Verpoorte R. (2009). Solvent derived artifacts in natural products chemistry. Nat. Prod. Commun..

[6] Bijauliya R.K., Alok S., Kumar M. (2017). A Comprehensive Review on Standardization of Herbal Drugs. Int. J. Pharm. Sci. Rev. Res..

[7] Ruberto G., Biondi D., Renda A. (1999). The composition of the volatile oil of Ferulago nodosa obtained by steam distillation and supercritical carbon dioxide extraction. Phytochem. Anal..

[8] Marongiu B., Piras A., Porcedda S. (2006). Comparative analysis of the oil and supercritical CO2 extract of Artemisia arborescens L. and Helichrysum splendidum (Thunb.) Less. Nat. Prod. Res..

[9] Ohno A., Kizu H., Tomimori T. (1996). Studies on Nepalese Crude Drugs. XXI. On the Diterpenoid Constituents of the Aerial Part of Scutellaria discolor COLEBR. Chem. Pharm. Bull..

[10] Wang B., Wang X.-L., Wang S.-Q., Shen T., Liu Y.-Q., Yuan H., Lou H.-X., Wang X.-N. (2013). Cytotoxic Clerodane Diterpenoids from the Leaves and Twigs of Casearia balansae. J. Nat. Prod..

[11] Shen L., Ji H.-F. (2012). The pharmacology of curcumin: Is it the degradation products?. Trends Mol. Med..

[12] Stanic Z. (2016). Curcumin, a Compound from Natural Sources, a True Scientific Challenge—A Review. Plant Foods Hum. Nutr..

[13] Connolly J.D., Harrison L.J., Rycroft D.S. (1984). The structure of tamariscol, a new pacifigorgiane sesquiterpenoid alcohol from the liverwort frullania tamarisci. Tetrahedron Lett..

[14] Connolly J.D. (1990). Monoterpenoids and Sesquiterpenoids from the Hepaticae. Bryophytes, Their Chemistry and Chemical Taxonomy.

[15] Asakawa Y., Sono M., Wakamatsu M., Kondo K., Hattori S., Mizutani M. (1991). Geographical distribution of tamariscol, a mossy odorous sesquiterpene alcohol, in the liverwort *Frullania tamarisci* and related species. Phytochemistry.

[16] Tori M. (1995). Studies on the Absolute Configuration of Some Liverwort Sesquiterpenoids. Bioactive Natural Products.

[17] Tori M., Sono M., Asakawa Y. (1990). Absolute configuration and synthesis of the liverwort sesquiterpene alcohol tamariscol. J. Chem. Soc. Perkin Trans. 1.

[18] Tori M., Sono M., Nishigaki Y., Nakashima K., Asakawa Y. (1991). Studies on the liverwort sesquiterpene alcohol tamariscol. Synthesis and absolute configuration. J. Chem. Soc. Perkin Trans. 1.

[19] Izac R.R., Poet S.E., Fenical W., Van Engen D., Clardy J. (1982). The structure of pacifigorgiol, an ichthyotoxic sesquiterpenoid from the pacific gorgonian coral pacifigorgia cf. adamsii. Tetrahedron Lett..

[20] Paul C., König W.A., Muhle H. (2001). Pacifigorgianes and tamariscene as constituents of *Frullania tamarisci* and *Valeriana officinalis*. Phytochemistry.

[21] Faulkner D.J. (1984). Marine natural products: Metabolites of marine algae and herbivorous marine molluscs. Nat. Prod. Rep..

[22] Iliopoulou D., Vagias C., Galanakis D., Argyropoulos D., Roussis V. (2002). Brasilane-type sesquiterpenoids from Laurencia obtusa. Org. Lett..

[23] Melching S., A König W. (1999). Sesquiterpenes from the essential oil of the liverwort *Conocephalum conicum*. Phytochemistry.

[24] Fulmer G.R., Miller A.J.M., Sherden N.H., Gottlieb H.E., Nudelman A., Stoltz B.M., Bercaw J.E., Goldberg K.I. (2010). NMR Chemical Shifts of Trace Impurities: Common Laboratory Solvents, Organics, and Gases in Deuterated Solvents Relevant to the Organometallic Chemist. Organometallics.

[25] Shamma M., Rahimizadeh M. (1986). The Identity of Chileninone with Berberrubine. The Problem of True Natural Products vs. Artifacts of Isolation. J. Nat. Prod..

[26] Pannequin A., Tintaru A., Desjobert J.-M., Costa J., Muselli A. (2017). New advances in the volatile metabolites of *Frullania tamarisci*. Flavour Fragr. J..

[27] Clayden J., Greeves N., Warren S.G. (2012). Organic chemistry.

[28] Tori M., Nakashima K., Asakawa Y., Connolly J.D., Harrison L.J., Rycroft D.S., Singh J., Woods N. (1995). Structure of conocephalenol, a sesquiterpenoid alcohol from the European liverwort *Conocephalum conicum*: Determination of the absolute configuration by total synthesis. J. Chem. Soc. Perkin Trans. 1.

[29] Berger S., Braun S. (2004). 200 and more NMR experiments: A practical course, 3rd rev. and expanded ed..

[30] Smith A.J.E., Smith R. (2004). The Moss Flora of Britain and Ireland.

[31] Matesanz R. (1997). The Council of Europe and organ transplantation. Transplant. Proc..

[32] Pannequin A. Caractérisation Chimique des Bryophytes de Corse et Propriétés Biologiques. Ph.D. Thesis.

[33] Frisch M.J., Trucks G.W., Schlegel H.B., Scuseria G.E., Robb M.A., Cheeseman J.R., Scalmani G., Barone V., Petersson G.A., Nakatsuji H. (2016). Gaussian 16 Rev. C.01.

[34] Cossi M., Scalmani G., Rega N., Barone V. (2002). New developments in the polarizable continuum model for quantum mechanical and classical calculations on molecules in solution. J. Chem. Phys..

[35] Dewar M.J.S., Healy E.F., Stewart J.J.P. (1984). Location of transition states in reaction mechanisms. J. Chem. Soc., Faraday Trans. 2.

