# Supplementary material for: Caution: Chemical Instability of Natural Biomolecules During Routine Analysis
Source: Molecules. 2020 Jul 20;25(14):3292. doi: 10.3390/molecules25143292 (PMC7397321; doi:10.3390/molecules25143292)
Supplement: Supplementary file 1 [file molecules-25-03292-s001.pdf]

Caution: Chemical Instability of Natural Biomolecules During Routine Analysis

Anaïs Pannequin [1], Erik Laurini [2], Laurent Giordano [3], Alain Muselli [1], Sabrina Pricl [2,4 §] and Aura Tintaru [5,*]

[1] Université de Corse, UMR CNRS 6134 SPE, Laboratoire Chimie des Produits Naturels (CPN), Campus Grimaldi, BP 52, 20250 Corte, France ; pannequin_a@univ-corse.fr (A.P.) ; muselli_a@univ-corse.fr (A.M.)
[2] Molecular Biology and Nanotechnology Laboratory (MolBNL@UniTS), DEA, University of Trieste, Italy; erik.laurini@dia.units.it (E.L.), sabrina.pricl@dia.units.it (S.P.)
[3] Aix Marseille Univ, CNRS, Ecole Centrale de Marseille, Institut des Sciences Moléculaires de Marseille UMR7313, 13397 Marseille, France ; laurent.giordano@centrale-marseille.fr
[4] Department of General Biophysics, Faculty of Biology and Environmental Protection, University of Lodz, Lodz, Poland
[5] Aix Marseille Univ, CNRS, Institut de Chimie Radicalaire UMR7273, 13397 Marseille, France; aura.tintaru@univ-amu.fr
* Correspondence: aura.tintaru@univ-amu.fr; Tel.: +33-491-28-8926 (A.T.); [§]Senior co-author

Supporting Information

Table S1. List of harvested *Frullania tamarisci* and *Conocephalum conicum* samples……………...… p.2

Table S2. Pacifigorgiane-like constituents present in the Corsican *Frullania tamarisci* extracts … p.2

Figure S1: Full ^{13}C-NMR spectra of tamariscol rich fraction acquired in $CDCl_3$ …………………… p.3

Table S3. Brasiladiene-like constituents present in the Corsican *C. conicum* extracts …………….. p.3

Figure S2: Full ^{13}C-NMR spectra of conocephalenol rich fraction acquired in $CDCl_3$ ……………… p.4

Figure S3: ^{1}H NMR recorded (500MHz, 300K) in $CDCl_3$ filtered on the basic alumina …………… p.5

Table S1. List of harvested *Frullania tamarisci* and *Conocephalum conicum* samples

Sample	Location	GPS Coodinates		Altitude (m)	Harvest Date (month-year)
		Frullania tamarisci			
**S1**					June-15
**S1.1**					February-16
**S1.2**	Restonica	9°07'45.9"E	42°17'18.0"N	400	March-16
**S1.3**					May-16
**S1.4**					June-16
**S2**	Vizzavona	9°06'18.8"E	42°05'52.6"N	1160	June-16
**S3**	Vivario	9°10'01.2"E	42°11'31.9"N	400	June-16
		Conocephalum conicum			
**S1**	Sant'andréa di Bozzio	9°17'41.96"E	42°17'51,03"	750	March-14
**S1.1**					June-15
**S2**					April-14
**S2.1**	Vizzavona	9°7'52.3"E	42°7' 33.41" N	1000 to 1100	June-15
**S2.2**					March-17
**S2.3**					January-18
**S3**	Corrano	9°3' 50.06" E	41°53' 35.86" N	320	April-15
**S3.1**					April-18
**S4**	Sorba	9°11' 24.89" E	41°58' 15.86" N	690	April-15
**S4.1**					April-18
**S5**	Frassetu	9°1' 14.33" E	41°53' 50.28" N	530	April-18

Table S2. Pacifigorgiane-like constituents detected in the Corsican *Frullania tamarisci* extracts

**Compound** [1]	**LRIa** [2]	**RIa** [3]	**RIp** [4]	% [5]			
				**EO**	**HY**	**MAC**	**SPME**
Pacifigorgia-1(9),10-diene (**5**)	1384	-	1526	0.13	-	3.80	1.50
Pacifigorgia-1,10-diene (**6**)	1400	1402	1517	0.91	-	0.18	5.80
Pacifigorgia-1(6),10-diene (**7**)	1414	1415	1538	0.88	-	-	4.50
Pacifigorgia-2,10-diene (**8**)	1422	1421	1553	0.90	-	1.00	6.10
Pacifigorgia-2(10),11-diene (**9**)	1435	1434	1658	0.73	-	0.97	3.50
Tamariscol (**1**)	1535	1533	1929	41.49	5.45	15.00	32.70
Pacifigorgiol (**2**)	1540	1533	1929	-	-	-	-

[1] Order of elution is given on apolar column (Rtx-1) [2]LRI_A: Literature retention indices on apolar column reported from literature [1]. [3]RI_A: Retention indices on Rtx-1 (apolar) column [4] RI_P: Retention indices on Rtx-Wax (polar) column [5]Percentages of individual components on Rtx-1 except pacifigorgiol with same Ri_a and Ri_P, this compound detected in NMR EO: Essential oil, HY: Hydrosol, MAC: diethyl oxide maceration, SPME: Solid Phase MicroExtraction

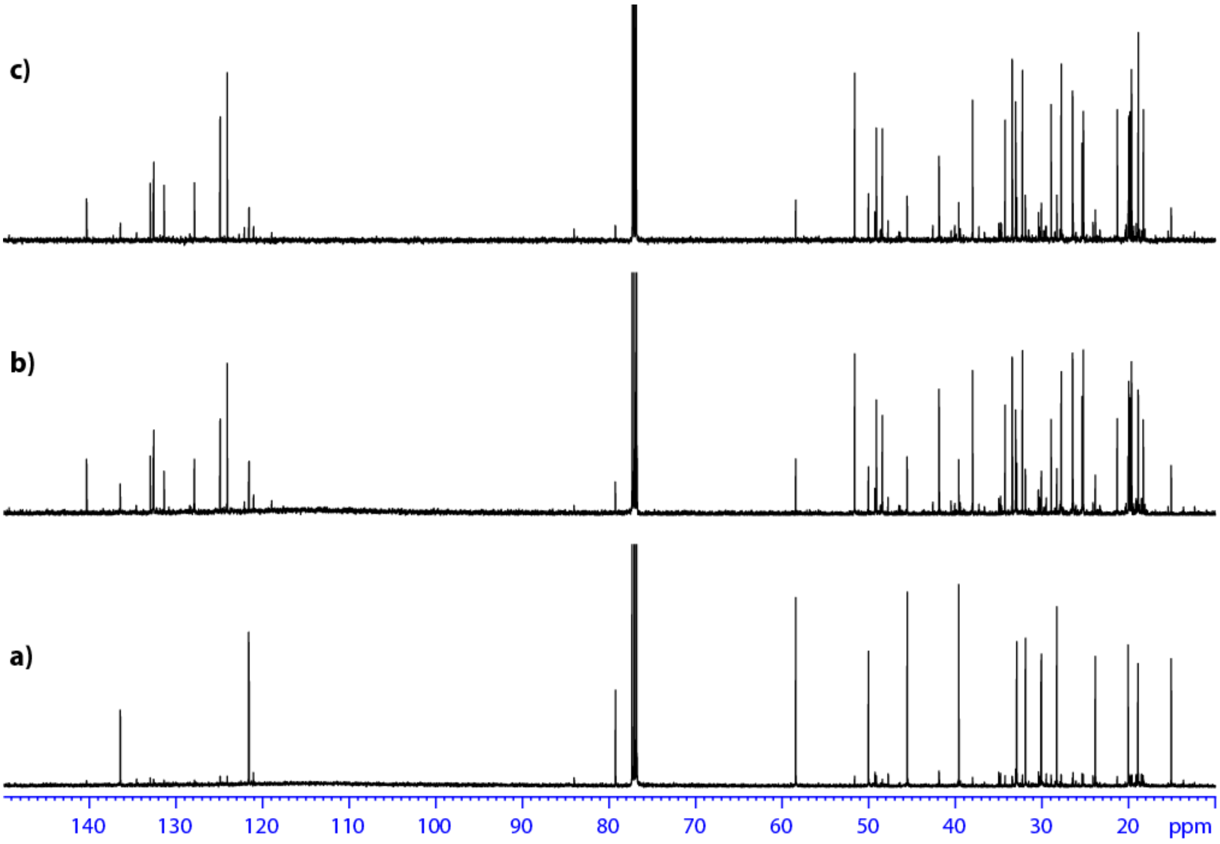

Figure S1. Full ^{13}C-NMR spectra of tamariscol rich fraction (>95%) acquired in $CDCl_3$ (150.90 MHz, 300K) at **a)** 30 min, **b)** 12h and **c)** 6 days respectively, after sample preparation.

Table S3. Brasiladiene-like constituents detected in the Corsican *C. conicum* extracts

**Compound** [1]	**LRIa** [2]	**RIa** [3]	**RIp** [4]	% [5]		
				**EO**	**MAC**	**SPME**
brasila-1,10-diene	1307	1305	1403	0,8	1,4	3,8
brasila-5,10-diene (**10**)/2	1335	1335	1495	5,4	2,4	5,3
brasila-5(10),6-diene (**12**)/4	1370	1372	1495	2,0	1,8	1,9
brasila-1(6),5(10)-diene (**11**)/3	1444	1441	1601	5,3	2,5	4,7
conocephalenol	1497	1492	1913	5,6	19,2	0,5
tamariscol	1533	1537	1929	0,4	-	-

[1] Order of elution is given on apolar column (Rtx-1) [2] LRIA: Literature retention indices on apolar column reported from literature [1]. [3]RIA: Retention indices on Rtx-1 (apolar) column [4] RIP: Retention indices on Rtx-Wax (polar) column. EO: Essential oil, HY: Hydrosol, MAC: diethyl oxide maceration, SPME: Solide phase MicroExtraction

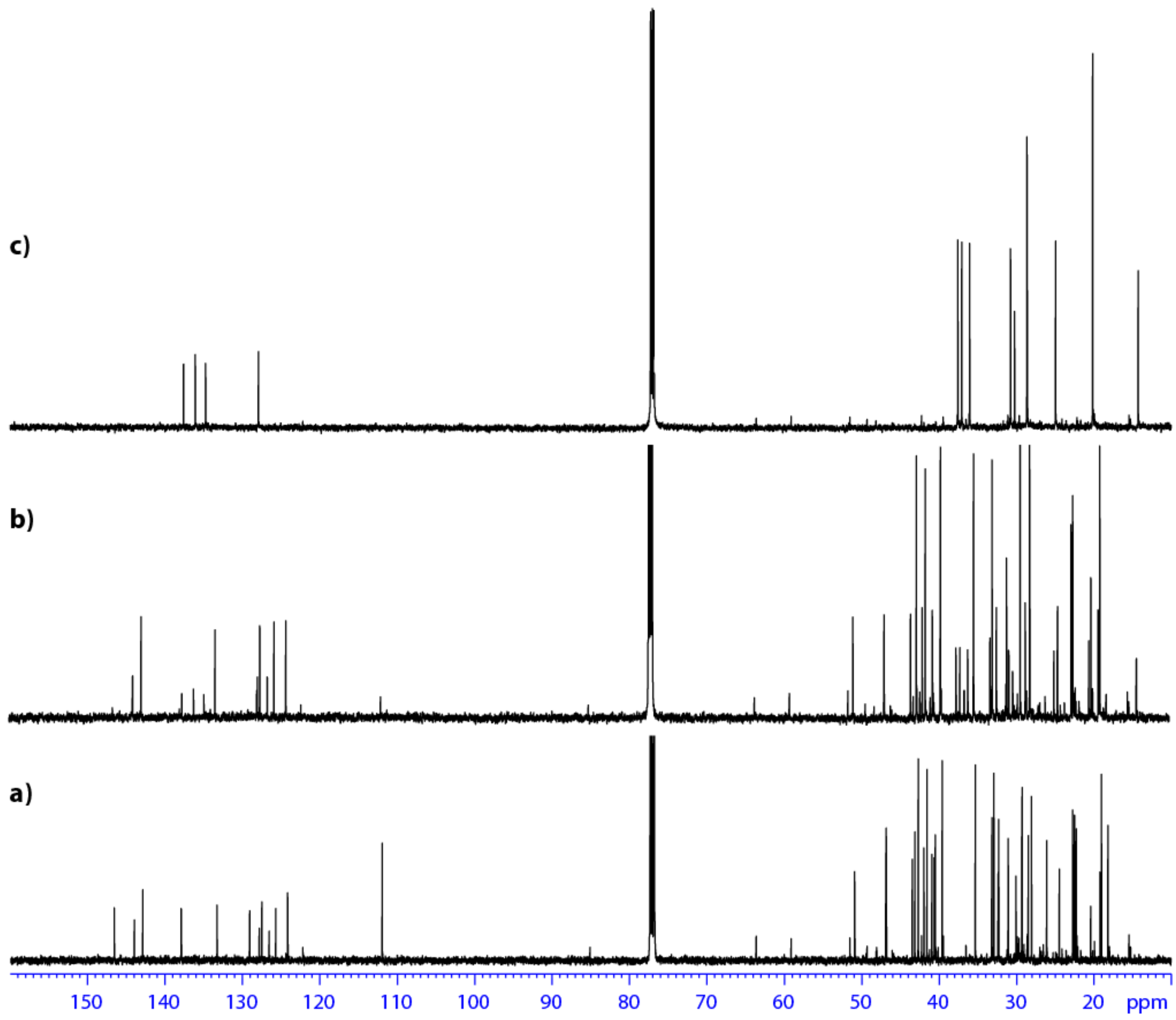

Figure S2. Full ^{13}C-NMR spectra of conocephalenol rich fraction (80%) acquired in $CDCl_3$ (150.90 MHz, 300K) at **a)** 30 min, **b)** 2h and **c)** 6h respectively, after sample preparation.

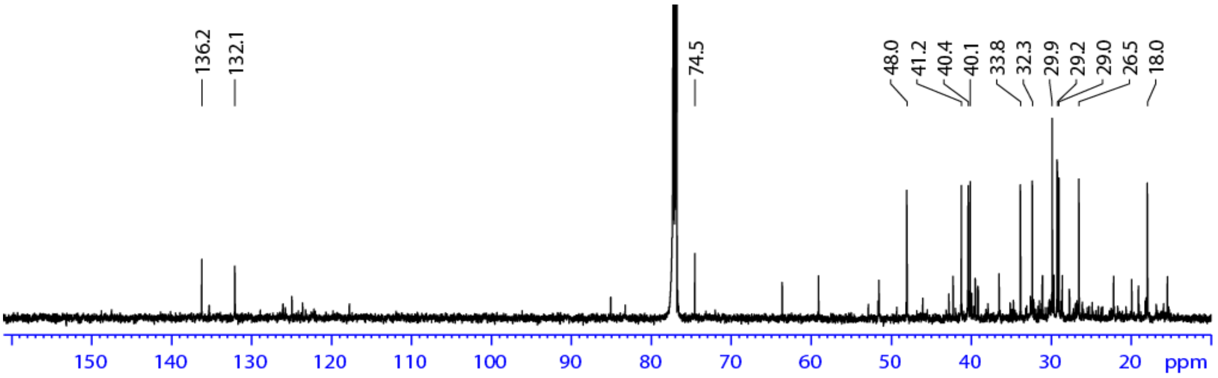

Figure S3: ^{1}H NMR recorded (500MHz, 300K) in $CDCl_3$ filtered on the basic alumine prior to sample dissolution. The labels are showing the obtained chemical shift of conocephalenol; the analyzed fraction contains also (-)-epi-Presilphiperfolan-1-ol [2].

Bibliography

[1] 'Terpenoids Library List - MassFinder'. https://massfinder.com/wiki/Terpenoids_Library_List (accessed May 13, 2019).

[2] S. C. Pinto *et al.*, '(−)-epi-Presilphiperfolan-1-ol, a new triquinane sesquiterpene from the essential oil of Anemia tomentosa var. anthriscifolia (Pteridophyta)', *Tetrahedron Lett.*, vol. 50, no. 33, pp. 4785–4787, Aug. 2009, doi: 10.1016/j.tetlet.2009.06.046.
